# Pasteurism in Russia

**Published:** 1887-02

**Authors:** 


### Medical News.

Pasteurism in Russia.—The operations of the Russian Pasteur Institute at Odessa have proved a signal failure. One third of all the patients treated have died, and it seems highly probable that some of the deaths are due solely to the treatment. The following case, from *Novoe Vremia*, will serve as an illustration: In the month of July, 1886, Arthur Stoboi was bitten by a dog supposed to be affected with rabies. The boy was immediately sent to the institute of Dr. Boniville, in Odessa, and was there treated according to the system of Pasteur. On the 9th of November, he experienced a severe pain in the place where the virus had been inocculated by the physician, and, two days afterward, died of rabies. The dog which bit the boy, however, is still alive, and has, up to the present time, shown no symptoms of hydrophobia. We must thus necessarily attribute the death to the treatment alone.

---

Widmark, in a recent examination of the eyes of school children in Sweden, has discovered that at the age of sixteen one-third of all the pupils both male and female are myopic. In the higher schools for young ladies more than half are myopic; in one school sixty-six per cent. had this defect of vision. He concludes:

1. That the percentage of myopics increases directly with the grade of the scholars the rate of increase being greater for females than males.

2. That the degree of myopics increases directly with the grade of the scholars, being also greater for females than for males.

